# A Recent Meeting at the New York Academy

**Published:** 1920-04-24

**Authors:** 


### THROMBOANGITIS OBLITERANS.

### A Recent Meeting at the New York Academy.

A type of lesion which has at all times attracted considerable attention, not only from its sinister meaning to the unfortunate patient, but also from its often obscure pathology and etiology, is that which may conveniently be termed pre-senile gangrene. In this short note it is impossible to deal with the whole list of well-recognised and possible causes of this condition, and it is proposed to consider chiefly that peculiar state now generally termed thromboangitis obliterans, which is relatively rarely met with, extremely difficult of diagnosis, and is repeatedly appearing in the literature as something of a medical conundrum. Often the disease is stated as occurring only in those of Jewish extraction, but upon this and other debatable points considerable light was thrown at a recent meeting of the New York Academy of Medicine, when an extended discussion followed excellent papers read by Drs. W. Meyer, Buerger, and A. Bernhard. Upon the notes of the meeting is based the major part of the present article, and, in their simpler form, it is hoped the explanations provided may be of use to the practitioner should he encounter one of these baffling cases.

### The Etiology.

In the first place it is explained that the disease is apparently of much more common occurrence in the New World than in the Old, and the general opinion is that the changes and thickening of the inner coats of the vessels are due to the action of some poison actually circulating in the blood. In the first paper, by Dr. Meyer, attempt is made to ascribe the whole syndrome to the use, or rather abuse, of tobacco. He explains that "The feature of tobacco abuse in these cases has already attracted the attention of many observers. But to my knowledge no one, so far, has undertaken to make the abuse of tobacco primarily responsible for the disease—to trace the development of its symptoms to long-continued and excessive cigarette smoking." He proceeds to divide tobacco poisoning into three stages: (*a*) The acute poisoning; (*b*) the long period of tolerance, during which absorption may ultimately exceed elimination and an accumulation of poison result; producing (*c*) a stage of saturation of the system. It is suggested that in this third stage the majority of thromboangitis obliterans cases appear to be found. The interval between successive poisonings may have been so short that a vicious circle is set up through depression of the sympathetic nervous system, causing deficient action of the excretory glands actuated thereby, producing in turn still further accumulation and progressive depression of the nerve complex. Generally it is agreed that the Jews are especially predisposed to the disease, and the author fits this fact in with his hypothesis by providing a reason on grounds of heredity. "But," he says, "inasmuch as not all those who abuse tobacco to the same extent, as our patients have done, are seized with thromboangitis obliterans, the disease, I believe, clearly demands for the onset of the vicious circle the presence of an additional factor. I look for it in a *predisposition of the nervous system*. As a matter of fact, all the patients seem to be thus predisposed. They are all Russian-Polish Hebrews who have immigrated from, or (exceptionally) whose ancestors immigrated from, a narrow belt stretching from the Baltic to the Black Sea. The centuries of persecution under which generations of these Jews have gone through life in a more or less constantly hysterical condition have left their imprint upon these people. They are neurasthenics by heredity."

### Some Exceptions.

On the other hand, it was claimed by other observers that the disease does occasionally occur in women who certainly did not smoke, whereupon two explanatory lines are open. Either the predisposition transmitted from parents may be sufficiently strong to allow endarteritis to occur from milder intoxications, or else the condition seen might not be true thromboangitis obliterans at all. After reading the views held by the various authorities who spoke at the meeting, it would appear that this particular type of gangrene can be, and mostly is, produced by a combination of excessive tobacco smoking, lack of compensating relaxation and rest, and a continued laborious existence. The predisposition seems to lie entirely with the Jews or their direct descendants, and in extreme cases the actual tobacco intoxication may not be necessary to start the arterial disease. The most important point in diagnosis seems, therefore, to lie in appreciation of the patient's nationality.

### Treatment.

In gangrenous conditions rational treatment calls for the obvious surgical measures, but otherwise experience shows that, once formed, nothing appears to cause the arterial condition to retrogress. In a sense, the only real cure for the disease is prophylaxis. Dr. Meyer is emphatic enough to say that people so constituted as the Hebrews *should not smoke*, but, in this country at least, with so rare a disease it would be not a little difficult to open an anti-tobacco campaign on these lines alone. However, we agree with the suggestion that the angitis, like intermittent limping, claudication, and the gangrene itself, is only one of the features of the disease, and not the disease itself. The initial pathology lies in all probability in the blood, and not in the vessels, so that if further investigation is made, with a view either to providing treatment or to locating a cause, it must be through the medium of the circulating blood and lymph; and that, although tobacco is a more than likely

**Thromboangitis Obliterans**—(*continued*).

instigator of the obliterative arterial changes, there must be other toxins capable of doing the same thing. The almost startling hereditary and racial factor seems to be of paramount importance, but is an aid to diagnosis rather than to therapeutic methods.

The Pathology.

The paper read by Dr. Buerger, who has done considerable recent investigational work in this disease, is of great interest. The name we have so far used in this note was, in fact, invented by him as more correct than endarteritis obliterans, to which we had become accustomed, and on this occasion, as previously, he confined himself to the purely pathological side of the subject.

He describes a healed stage where the thrombotic occlusion of the vessels, both arteries and veins, has given way to organisation, canalisation, and connective tissue formation in the occluding clot, with conversion of the arteries into hard cords. In this stage a variety of permanent symptom complexes may be produced, but he was emphatic that many of the individual lesions producing them are healed as far as the active disease is concerned. Investigation of pathological origin in such old cases is well-nigh hopeless.

A Noteworthy Acute Stage.

On the other hand, an acute stage is noted in which the process of toxin invasion is progressing, and an excellent series of sections illustrates that there is an initial active and rapid inflammatory process of both arteries and veins. Therefore we see a process which presents marked contrasts to the gradual changes which we customarily associate with either arteriosclerotic changes or the different varieties of thrombus production.

The best way to present the situation as it now stands is to adopt the author's summary of his investigational findings. Briefly these are: (1) Thromboangitis obliterans consists of acute inflammatory lesions and occlusive thrombosis of the arteries and veins in a characteristic sequence. (2) The first symptoms are produced mainly by early local thrombotic occlusion. (3) This thrombosis is probably preceded, and certainly accompanied, by an acute local inflammatory or exudative stage. (4) Both deep and superficial vessels are involved, and the lesions are the same and characteristic in both. (5) The histology of the exudate and thrombus formation point to an acute infectious process. (6) Finally, the development of fibrotic tissue in the adventitia of the vessels binds together the associated artery, vein, and nerves, with the typical result seen in an advanced gangrenous case.

Future Research.

It is unnecessary to remark that all known infecting agents have been searched for in vain—*e.g.*, spirochætes, tubercle bacilli, the common pyogenic organisms by cultures and in sections, but the general feeling of the meeting appears to have been that the best hope of completing the pathology of the disease lies in yet further examination of the " acute " material from affected vessels. There are obvious difficulties in the way of obtaining such specimens, and they have been as yet relatively infrequently available, but it seems that one great step at least has been taken in recognising the direct infective nature of the lesions; and we hope in the near future to hear that the workers in this section of research have discovered at least the nature of the toxic substance, and so brought further medical power to bear against a disease which is interesting, not only in itself but in the possible light which mastery of it might throw upon the nature of other as yet unknown infecting agents.

